# Acetone vapour-assisted growth of 2D single-crystalline organic lead halide perovskite microplates and their temperature-enhanced photoluminescence†

**DOI:** 10.1039/c8ra00583d

**Published:** 2018-04-18

**Authors:** Wenhao Zhai, Chaoyang Ge, Xin Fang, Kun Zhang, Cheng Tian, Kai Yuan, Shuren Sun, Yanping Li, Weixi Chen, Guangzhao Ran

**Affiliations:** State Key Laboratory for Artificial Microstructure and Mesoscopic Physics, School of Physics, Peking University Beijing 100871 China rangz@pku.edu.cn

## Abstract

We adopt an acetone vapour-assisted method to grow high quality single-crystalline microplates of two-dimensional (2D) perovskite, 2-phenylethylammonium lead bromide [(C_6_H_5_C_2_H_4_NH_3_)_2_PbBr_4_]. The microplates, converted from the spin-coated films, are well-defined rectangles. Temperature dependent photoluminescence (PL) spectroscopy shows that the band gap PL is enhanced markedly with increasing temperature up to 218 K, accompanied by the quenching of the PL related to the trap states, which perhaps results from the exciton–phonon couplings. The optical phonon energy around 50 meV and the exciton binding energy around 120 meV are derived by fitting the band gap PL linewidths and intensities at different temperatures, respectively.

## Abstract 2

We report an acetone vapour-assisted method to grow single-crystalline 2D perovskite microplates and find their temperature-enhanced photoluminescence.

## Graphical abstract

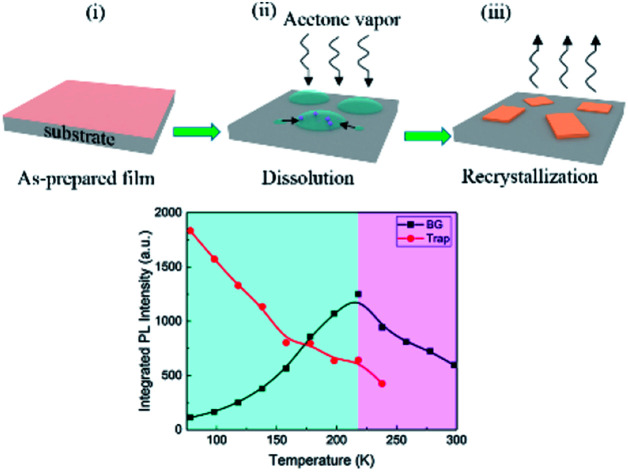

## Introduction

Organic lead halide perovskites have gained significant attention owing to the low-cost solution process and their promising application prospects in light harvesting^1–4^ and light emission.^5–7^ Nowadays, perovskites go into two-dimensional (2D) form for their tailorable electronic structures and optical properties. Such a 2D perovskite adopts a layered structure, a natural quantum-well structure.^8^ When it is extremely thin, each layer is only composed of an inorganic atomic layer (“well”) of PbX_4_^2−^ (X = Cl, Br, or I) octahedra, capped with two layers of organic long chain ammonium cations on both sides (“barrier”). The adjacent layers are held together by weak van der Waals forces. These unique structures enhance electron–hole interactions and result in large exciton binding energy,^9^ benefitting efficient photoluminescence (PL). While in 2D perovskites, strong exciton–phonon coupling usually exists and causes nonradiative exciton relaxation,^10^ which should be suppressed in light emitting applications.^11,12^

Several methods^13–17^ have been developed to synthesize 2D perovskites. In the earlier work, the 2D perovskites were spin-coated polycrystalline films, but the ultimate performances of the resulting devices were limited by extensive disorder in polycrystalline films, so they only worked at low temperature.^13,18^ To improve the crystal quality and synthesize single-crystalline 2D perovskites, Dou *et. al.* adopted ternary co-solvent method to grow atomically thin single-crystalline microsheets of 2D perovskite (C_4_H_9_NH_3_)_2_PbBr_4_.^16^ Ma *et. al.* used solution-phase transport method to synthesize single-crystalline microplates of (PEA)_2_PbBr_4_ with well-defined rectangular geometry,^17^ where PEA stands for C_6_H_5_C_2_H_4_NH_3_. Obviously, the single crystalline 2D perovskites are hard to achieve, and their growth has only achieved a limited success so far. In this work, we have developed an acetone vapour-assisted method to convert the spin-coated 2D perovskite (PEA)_2_PbBr_4_ thin films into well-defined single-crystalline microplates, which have efficient purple-blue emission at room temperature. We further investigate their growth process and temperature dependent PL from both the band gap transition and trap states, and find their anomalous temperature-dependent PL behaviours resulting from the exciton–phonon couplings. Optical phonon energy and exciton binding energy have been obtained.

## Experimental

**Fig. 1:**
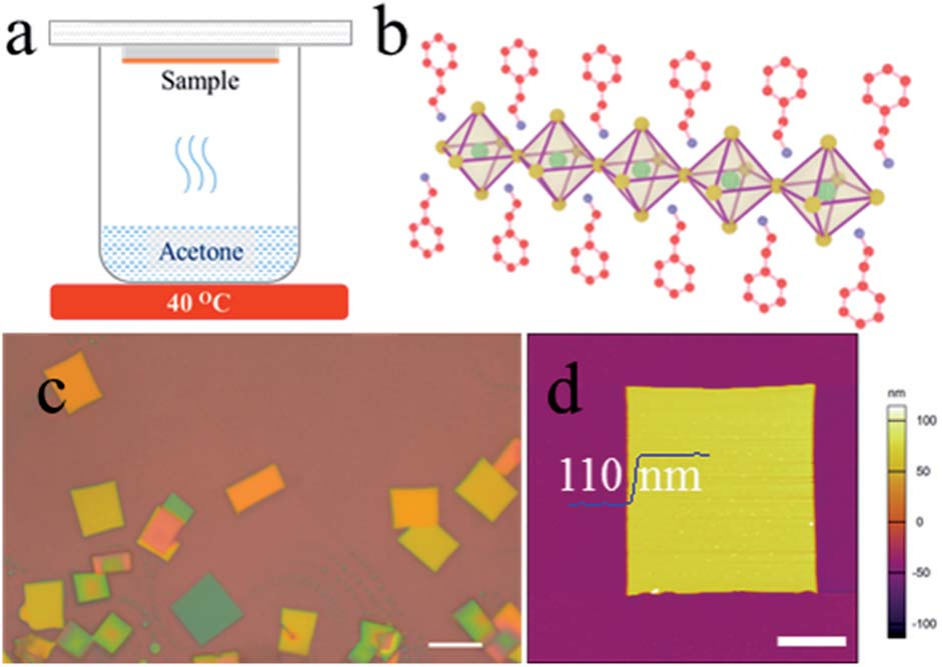
(a) Schematic diagram of the synthesis apparatus. (b) Structural illustration of a single layer (PEA)_2_PbBr_4_. The inorganic layer is made up of PbBr_4_^2−^ octahedra and the organic capping layers are 2-phenylethylammonium. The green, yellow, red and blue solid balls represent lead (Pb), bromide (Br), carbon (C), nitrogen (N) atoms, respectively. The hydrogen atoms are neglected. (c) Optical image of (PEA)_2_PbBr_4_ microplates. The scale bar is 10 μm. (d) AFM image of a typical (PEA)_2_PbBr_4_ microplate. The scale bar is 5 μm.

The acetone vapour-assisted method for growing 2D perovskites (PEA)_2_PbBr_4_ microplates is depicted as follows. First, the 2D (PEA)_2_PbBr_4_ perovskite precursor solution (0.01 M) is prepared by mixing PEABr and PbBr_2_ in a molar ratio of 2 : 1 in *N*,*N*-dimethylformamide (DMF). The solution is spin-coated on air-plasma treated SiO_2_/Si substrates with a speed of 4000 rpm for 60 s to form thin films, which are not heated to remove the residual DMF. Then, the samples are stuck to the bottom of a glass Petri dish (*Φ* 60 mm) and placed face-down in the acetone vapour at 40 °C for 1 to 5 min as shown in Fig. 1a. Here acetone (8 mL) is contained in a 25 mL beaker and the distance between the acetone and samples is about 1 cm. Finally, the samples are annealed at 70 °C for 10 min. All processes are performed in a nitrogen-filled glovebox.

## Results and discussion

A structural illustration of a single layer (PEA)_2_PbBr_4_ is drawn in Fig. 1b. An optical image of the typical (PEA)_2_PbBr_4_ microplates is shown in Fig. 1c. As can be seen, the microplates have a uniform rectangle shape with a size of ∼10 μm. Atomic force microscopy (AFM) image shows that their typical thickness is around 110 nm in Fig. 1d. The statistical data of the sizes and thicknesses of 2D perovskite microplates are shown in Fig. S1.†

**Fig. 2:**
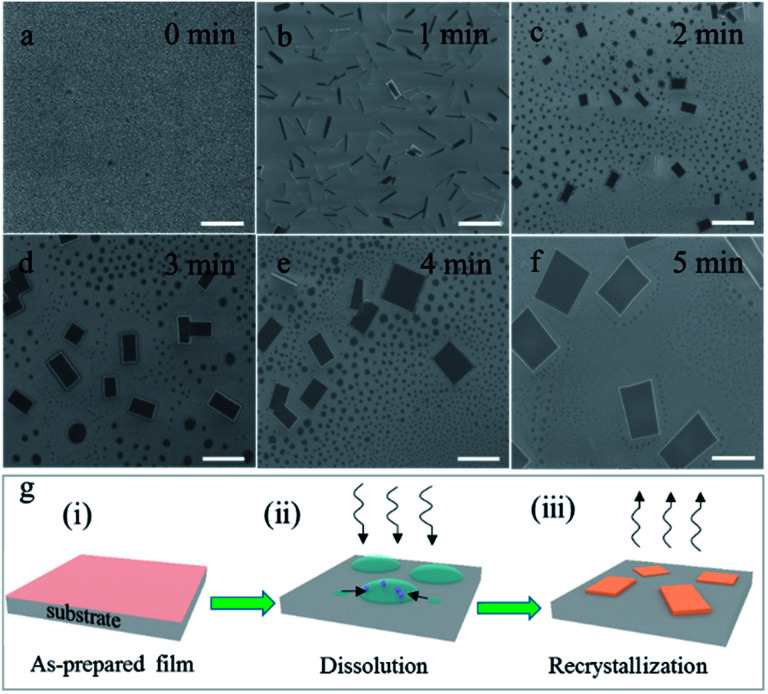
Scanning electron microscope (SEM) images indicated the growth process of (PEA)_2_PbBr_4_ microplates. (a) (PEA)_2_PbBr_4_ thin film without acetone vapour treatment. (b–f) (PEA)_2_PbBr_4_ microcrystals treated with acetone vapour at 40 °C for 1 to 5 min, respectively. All scale bars are 5 μm. (g) Schematic diagram of the acetone-assisted growth process of 2D perovskite microplates. The wavy arrows represent the acetone vapour. The sample in (ii) has been turned over for clarity.

To probe the detailed growth process, the (PEA)_2_PbBr_4_ perovskite films were treated by acetone for different durations and at different heating temperatures. We first optimized the temperature of acetone to be 40 °C. Thin films shown in Fig. 2a become needle-shaped crystals as shown in Fig. 2b when treated by acetone at 40 °C for 1 min. As time increases from 2 to 5 min, 2D perovskites microplates are formed and grow up in size gradually as shown in Fig. 2c–f, respectively. X-ray diffraction (XRD) patterns (see Fig. S2†) show that the linewidths of the microplate samples are much narrower than those of the film samples are. For the (001) patterns, their linewidths are 0.0669° and 0.2676°, respectively, indicating much better crystallinity of the microplates. The growth process above can be explained by a dissolution–recrystallization mechanism, which is distinguished from the anti-solvent diffusion–crystallization mechanism that vapour diffuses into the solution of the precursors and promotes the crystallization of the perovskites.^19,20^ In our experiment, acetone vapour is emitted from mildly heated acetone at 40 °C and condenses into little liquid droplets when meeting the sample of perovskite film, and the thin film begins to redissolve in acetone, which can be seen by naked eyes. Although the solubility of (PEA)_2_PbBr_4_ perovskite in acetone is extremely low, the amount of acetone is much excessive relative to perovskite in this experiment. When the samples are turned over, the perovskites easily recrystallize to form microplates, as schematically illustrated in Fig. 2g. Here, the acetone seems to play the role of a “solvent” for the perovskites, rather than an “anti-solvent”. Noticeably, at room temperature, the products are needle-shaped crystals, and microplates are scarcely found in Fig. S3.†

**Fig. 3:**
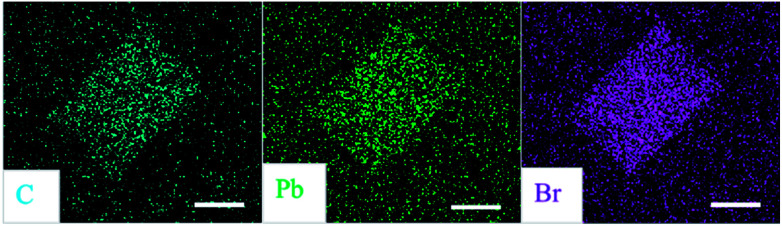
EDS mapping of individual (PEA)_2_PbBr_4_ microplate. All scale bars are 10 μm.

Energy-dispersive X-ray spectroscopy (EDS) is performed to investigate the composition of thin film and individual microplate and the results are summarized in Table 1. The element ratios Br/Pb of film and microplate are both close to 4 : 1, in accordance with the stoichiometry of 2D perovskites (PEA)_2_PbBr_4_. In Fig. 3, EDS mapping shows the homogeneous elemental distributions of carbon, lead and bromine in a typical microplate, indicating that the perovskite molecules around the plate have mostly been dissolved, then transported and finally assembled into the plate during the growth process.

**Table 1:** Element ratio Br/Pb of 2D perovskite film and microplate by EDS analysis

2D perovskite	Element ratio Br/Pb
Film	4.02
Microplate	3.97

**Fig. 4:**
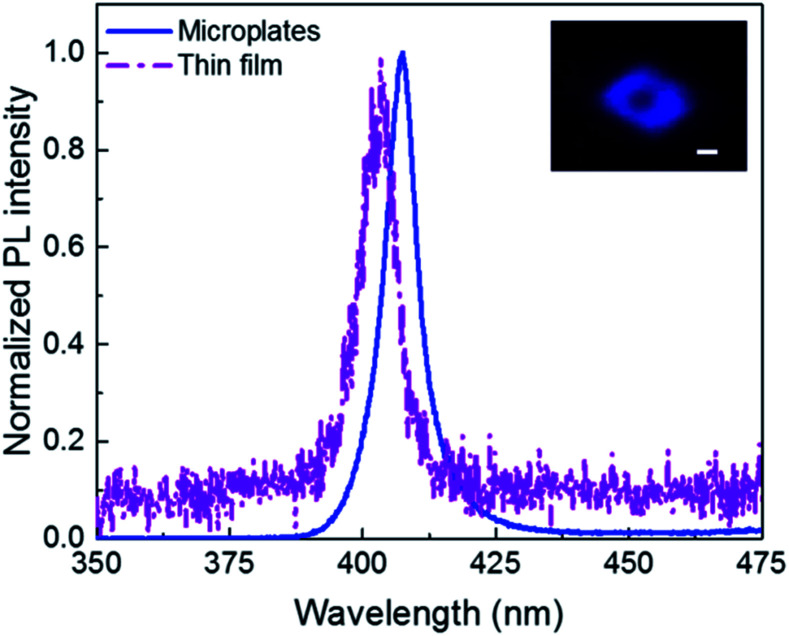
PL spectra of the (PEA)_2_PbBr_4_ microplates (blue line) and thin film (purple dash dot) at room temperature. Inset: the fluorescence image of a typical (PEA)_2_PbBr_4_ microplate. Scale bar is 5 μm.

It is well-known that the 2D perovskites exhibit high PL quantum yield and colour purity due to their self-organized quantum wells structure.^8^ PL properties of the 2D perovskites are investigated with 325 nm laser excitation. As shown in Fig. 4, (PEA)_2_PbBr_4_ microplates have a single peak PL spectrum at room temperature corresponding to the band gap transition. The peak locates at 407 nm and the full width at half maximum (FWHM) is 11 nm, showing a high colour purity, which is attractive for lasing and light emitting applications. The PL intensity of (PEA)_2_PbBr_4_ microplates is much higher than that of the thin film at an identical excitation condition (not shown here, but can be inferred from the lower signal-noise ratio for the film sample), indicating that improving the crystallinity of 2D perovskites (PEA)_2_PbBr_4_ leads to higher-efficiency PL. The reasons for that can be tentatively attributed to the decrease of the defect density and the suppression of scattering from polar optical phonons.^10^ The PL peak for the thin film sample locates at 403 nm, showing a slight blue shift relative to the single-crystalline sample. The inset is the fluorescence image of a typical (PEA)_2_PbBr_4_ microplate. We clearly observe the purple-blue light emission from the individual (PEA)_2_PbBr_4_ microplate.

**Fig. 5:**
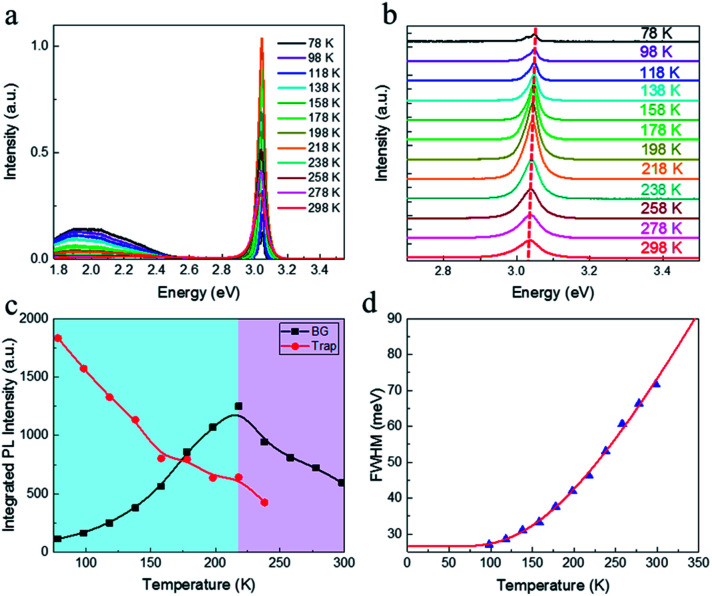
(a) Temperature dependent PL spectra from 78 to 298 K. (b) A magnification of temperature dependent PL spectra ranging from 2.7 to 3.5 eV. (c) Integrated PL intensity of band gap (BG) emission (black) and trap state emission (red). It should be mentioned that the PL spectra of trap states are firstly fitted by Gaussian function and the integrated PL intensities are calculated from the fitted curves. (d) Measured FWHMs (blue solid triangles) as a function of temperature and fitting result (red curve).

Temperature dependent PL spectroscopy is an effective tool to study the excitonic traps and phonon properties of semiconductors.^9,21–24^ Here, we present the temperature dependent PL spectra of (PEA)_2_PbBr_4_ microplates from liquid nitrogen temperature (78 K) to room temperature (298 K) in Fig. 5a. A magnification of the spectra ranging from 2.7 to 3.5 eV is shown in Fig. 5b, which indicates that the band gap peaks slightly red shift as temperature increases, a similar behaviour compared to most semiconductors. Moreover, a new peak emerges in the PL spectrum at 78 K, which will be discussed later. Noticeably, a broad emission with FWHM of a few hundred of meV is found in the low energy region of Fig. 5a at low temperatures, of which the intensity decreases with ascending temperature and becomes undetectable above 238 K (see Fig. S4a†). The presence of the broad emission can be ascribed to trap states in (PEA)_2_PbBr_4_, resulting from the electron-phonon coupling at the surface, which is similar to that reported in (RNH_3_)_2_PbI_4_ ^22^ and that in (HIS)PbBr_4_ (HIS = histammonium, 4-(2-ammonioethyl)-1*H*-imidazol-3-ium).^25^ As contrast, the band gap PL intensity increases markedly when temperature increases until up to 218 K, as summarized in Fig. 5c. Importantly, such behaviour, termed as temperature-enhanced PL, is opposite to most reported perovskite materials,^26–30^ which is only recently found in 2D perovskite (PBA)_2_PbI_4_ (PBA = 4-phenyl-1-butylammonium),^31^ similar to that in some inorganic semiconductors materials.^32–35^ Above this critical temperature, the band gap PL intensity begins to decrease. The ratio, *I*_BG_/*I*_Trap_, calculated from integrated intensity of band gap (BG) emission to that of trap-state emission at different temperature, as shown in Fig. S4b,† increases quickly with ascending temperature from 78 to 238 K, indicating that the trapped excitons can be activated into band gap excitons as temperature increases, which is much helpful for efficient band–band emission. The linewidth or FWHM as a function of temperature is plotted in Fig. 5d and the data are fitted by the following formula^36^ under the assumption that the broadening of the linewidth is mainly attributed to exciton–phonon interactions
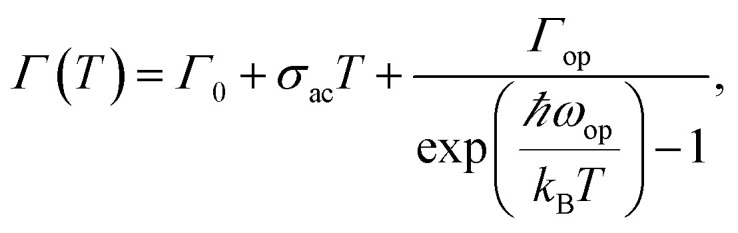
where *Γ*_0_ (meV) is the linewidth at 0 K, *σ*_ac_ (meV K^−1^) and *Γ*_op_ (meV) are the contributions of exciton–acoustic phonon interaction and exciton–optical phonon interaction, respectively, and *ħω*_op_ (meV) is the optical phonon energy. By fitting the results, *ħω*_op_ is found to be (50 ± 3) meV (see Table S1†). This involved phonon energy cannot be assigned by the Raman spectrum (shown in Fig. S5†) directly. It is much higher than the single phonon energy (below 150 cm^−1^) indicated by the Raman spectrum, consistent with the previous result.^27^ The electron–phonon coupling strength is fitted to be 276 meV, which is evidently larger than the reported value for the CsPbBr_3_ quantum dots,^37^ usually causing nonradiative decay for excitons.^10^ By simply fitting the PL quenching region (238–298 K) shown in Fig. 5c according to the Arrhenius formula,^38^ we have tentatively derived the exciton binding energy to be ∼120 meV, proving the existence of the room temperature excitons. Because no exciton binding energy for (PEA)_2_PbBr_4_ has been reported so far to our knowledge, we compare this value with that of other reported similar 2D materials.^39–41^ Our data have the same order of magnitude as the reported.

As mentioned before, the PL spectrum at 78 K of the single crystalline microplate is featured by a double peak structure, as shown in Fig. S6.† The new sharp peak is at 410 nm, the low energy side of the band gap peak. Besides, a weak shoulder around 415 nm at 78 K is also observable. A PL spectrum of the film sample is shown for comparison. However, the origins of the new peak and shoulder are not very clear so far. Several possible mechanisms^22–24^ such as phase transition, trapped exciton and multiple-exciton effects, await to be discriminated in the further study.

## Conclusions

In summary, uniform rectangle microplates of 2D perovskites (PEA)_2_PbBr_4_ have been synthesized by converting the thin films through the dissolution–recrystallization process in acetone vapour. Efficient purple-blue emission is observed with a narrow linewidth (∼11 nm). These single-crystalline 2D perovskite microplates have a unique temperature dependent PL behaviour. The optical phonon energy around 50 meV and the exciton binding energy around 120 meV are estimated by fitting the temperature dependent FWHMs and intensities, respectively.

## Conflicts of interest

There are no conflicts to declare.

## Supplementary Material

RA-008-C8RA00583D-s001

## References

[cit1] Lee M. M., Teuscher J., Miyasaka T., Murakami T. N., Snaith H. J. (2012). Science.

[cit2] Burschka J., Pellet N., Moon S.-J., Humphry-Baker R., Gao P., Nazeeruddin M. K., Grätzel M. (2013). Nature.

[cit3] Zhou H., Chen Q., Li G., Luo S., Song T.-b., Duan H.-S., Hong Z., You J., Liu Y., Yang Y. (2014). Science.

[cit4] Yang W. S., Park B.-W., Jung E. H., Jeon N. J., Kim Y. C., Lee D. U., Shin S. S., Seo J., Kim E. K., Noh J. H., Seok S. I. (2017). Science.

[cit5] Tan Z.-K., Moghaddam R. S., Lai M. L., Docampo P., Higler R., Deschler F., Price M., Sadhanala A., Pazos L. M., Credgington D., Hanusch F., Bein T., Snaith H. J., Friend R. H. (2014). Nat. Nanotechnol..

[cit6] Cho H., Jeong S.-H., Park M.-H., Kim Y.-H., Wolf C., Lee C.-L., Heo J. H., Sadhanala A., Myoung N., Yoo S., Im S. H., Friend R. H., Lee T.-W. (2015). Science.

[cit7] Wang N., Cheng L., Ge R., Zhang S., Miao Y., Zou W., Yi C., Sun Y., Cao Y., Yang R., Wei Y., Guo Q., Ke Y., Yu M., Jin Y., Liu Y., Ding Q., Di D., Yang L., Xing G., Tian H., Jin C., Gao F., Friend R. H., Wang J., Huang W. (2016). Nat. Photonics.

[cit8] Veldhuis S. A., Boix P. P., Yantara N., Li M., Sum T. C., Mathews N., Mhaisalkar S. G. (2016). Adv. Mater..

[cit9] Gauthron K., Lauret J. S., Doyennette L., Lanty G., Al Choueiry A., Zhang S. J., Brehier A., Largeau L., Mauguin O., Bloch J., Deleporte E. (2010). Opt. Express.

[cit10] Guo Z., Wu X., Zhu T., Zhu X., Huang L. (2016). ACS Nano.

[cit11] Dohner E. R., Hoke E. T., Karunadasa H. I. (2014). J. Am. Chem. Soc..

[cit12] Liang D., Peng Y., Fu Y., Shearer M. J., Zhang J., Zhai J., Zhang Y., Hamers R. J., Andrew T. L., Jin S. (2016). ACS Nano.

[cit13] Era M., Morimoto S., Tsutsui T., Saito S. (1994). Appl. Phys. Lett..

[cit14] Chondroudis K., Mitzi D. B. (1999). Chem. Mater..

[cit15] Mitzi D. B. (2001). Chem. Mater..

[cit16] Dou L., Wong A. B., Yu Y., Lai M., Kornienko N., Eaton S. W., Fu A., Bischak C. G., Ma J., Ding T., Ginsberg N. S., Wang L.-W., Alivisatos A. P., Yang P. (2015). Science.

[cit17] Ma D., Fu Y., Dang L., Zhai J., Guzei I. A., Jin S. (2017). Nano Res..

[cit18] Kondo T., Azuma T., Yuasa T., Ito R. (1998). Solid State Commun..

[cit19] Shi D., Adinolfi V., Comin R., Yuan M., Alarousu E., Buin A., Chen Y., Hoogland S., Rothenberger A., Katsiev K., Losovyj Y., Zhang X., Dowben P. A., Mohammed O. F., Sargent E. H., Bakr O. M. (2015). Science.

[cit20] Yuan Z., Zhou C., Tian Y., Shu Y., Messier J., Wang J. C., van de Burgt L. J., Kountouriotis K., Xin Y., Holt E., Schanze K., Clark R., Siegrist T., Ma B. (2017). Nat. Commun..

[cit21] Dammak T., Koubaa M., Boukheddaden K., Bougzhala H., Mlayah A., Abid Y. (2009). J. Phys. Chem. C.

[cit22] Wu X., Trinh M. T., Niesner D., Zhu H., Norman Z., Owen J. S., Yaffe O., Kudisch B. J., Zhu X. Y. (2015). J. Am. Chem. Soc..

[cit23] Yangui A., Pillet S., Mlayah A., Lusson A., Bouchez G., Triki S., Abid Y., Boukheddaden K. (2015). J. Chem. Phys..

[cit24] Gan L., He H., Li S., Li J., Ye Z. (2016). J. Mater. Chem. C.

[cit25] Smith M. D., Jaffe A., Dohner E. R., Lindenberg A. M., Karunadasa H. I. (2017). Chem. Sci..

[cit26] Ji H., Shi Z., Sun X., Li Y., Li S., Lei L., Wu D., Xu T., Li X., Du G. (2017). ACS Appl. Mater. Interfaces.

[cit27] Ai B., Liu C., Deng Z., Wang J., Han J., Zhao X. (2017). Phys. Chem. Chem. Phys..

[cit28] Zheng H., Dai J., Duan J., Chen F., Zhu G., Wang F., Xu C. (2017). J. Mater. Chem. C.

[cit29] Sarang S., Bonabi Naghadeh S., Luo B., Kumar P., Betady E., Tung V., Scheibner M., Zhang J. Z., Ghosh S. (2017). J. Phys. Chem. Lett..

[cit30] De Bastiani M., Dursun I., Zhang Y., Alshankiti B. A., Miao X.-H., Yin J., Yengel E., Alarousu E., Turedi B., Almutlaq J. M., Saidaminov M. I., Mitra S., Gereige I., AlSaggaf A., Zhu Y., Han Y., Roqan I. S., Bredas J.-L., Mohammed O. F., Bakr O. M. (2017). Chem. Mater..

[cit31] Gan L., Li J., Fang Z., He H., Ye Z. (2017). J. Phys. Chem. Lett..

[cit32] Gao J., Johnson J. C. (2012). ACS Nano.

[cit33] Turyanska L., Patanè A., Henini M., Hennequin B., Thomas N. R. (2007). Appl. Phys. Lett..

[cit34] Bogardus E. H., Bebb H. B. (1968). Phys. Rev..

[cit35] Wu K., He H., Lu Y., Huang J., Ye Z. (2012). Solid State Commun..

[cit36] Rudin S., Reinecke T. L., Segall B. (1990). Phys. Rev. B: Condens. Matter Mater. Phys..

[cit37] Shi Z., Li Y., Zhang Y., Chen Y., Li X., Wu D., Xu T., Shan C., Du G. (2017). Nano Lett..

[cit38] Wu K., Bera A., Ma C., Du Y., Yang Y., Li L., Wu T. (2014). Phys. Chem. Chem. Phys..

[cit39] Li J., Luo L., Huang H., Ma C., Ye Z., Zeng J., He H. (2017). J. Phys. Chem. Lett..

[cit40] Amerling E., Baniya S., Lafalce E., Zhang C., Vardeny Z. V., Whittaker-Brooks L. (2017). J. Phys. Chem. Lett..

[cit41] Blancon J.-C., Tsai H., Nie W., Stoumpos C. C., Pedesseau L., Katan C., Kepenekian M., Soe C. M. M., Appavoo K., Sfeir M. Y., Tretiak S., Ajayan P. M., Kanatzidis M. G., Even J., Crochet J. J., Mohite A. D. (2017). Science.

