# Supplementary material for: Acetone vapour-assisted growth of 2D single-crystalline organic lead halide perovskite microplates and their temperature-enhanced photoluminescence
Source: RSC Adv. 2018 Apr 18;8(26):14527–31. doi: 10.1039/c8ra00583d (PMC9079928; doi:10.1039/c8ra00583d)
Supplement: RA-008-C8RA00583D-s001 [file RA-008-C8RA00583D-s001.pdf]

Electronic Supplementary Material (ESI) for RSC Advances.
This journal is © The Royal Society of Chemistry 2018

Supporting Information for

Acetone vapor-assisted growth of 2D single-crystalline organic lead halide perovskite microplates and their temperature-enhanced photoluminescence

*Wenhao Zhai, Chaoyang Ge, Xin Fang, Kun Zhang, Cheng Tian, Kai Yuan, Shuren Sun, Yanping Li, Weixi Chen, and Guangzhao Ran**

State Key Laboratory for Artificial Microstructure and Mesoscopic Physics, School of Physics, Peking University, Beijing 100871, China

Corresponding author: rangz@pku.edu.cn

Materials

PEABr (99.5%) and $PbBr_2$ (99.99%) are purchased from Xi'an Polymer Light Technology Corporation. N, N-dimethylformamide (chromatographic grade) is purchased from Aladdin. Acetone (analytical grade) is purchased from Beijing Tong Guang Fine Chemicals Company. All reagents are used as received without further purification.

Characterizations

Optical microscope images are obtained with a Zeiss Microscope (Axio Imager.A2m). Atomic force microscopy (AFM) is performed on Cypher S AFM (Asylum Research) in tapping mode. Scanning electron microscope (SEM) images are acquired on a high resolution field emission SEM (FEI NanoSEM 430) with an

accelerating voltage of 5 kV. Energy-dispersive X-ray spectroscopy (EDS) and EDS mapping are also performed on the SEM with an EDS detector operating at 15 kV. X-ray Diffraction (XRD) patterns are measured using X-ray diffraction platform (model PANalytical X'Pert Pro) with a Cu-Kα radiation source (λ = 0.1541874 nm) at 40 kV and 40 mA. The angle accuracy is within ±0.0025 °. Steady state photoluminescence (PL) spectroscopy is performed on an integrated Raman/PL system (Horiba JY HR800) with an Olympus 15X NUV lens excited by a 325 nm helium-cadmium (He-Cd) laser. Temperature dependent PL spectroscopy is also measured on the Raman/PL system in a liquid N_2 purged cryostage (Linkam THMS 600) with the temperature ranging from 78 to 298 K. Raman spectroscopy is obtained from the Raman/PL system with an Olympus 100X lens excited by a 473 nm laser.

XRD patterns, SEM images, PL spectra and Raman spectroscopy

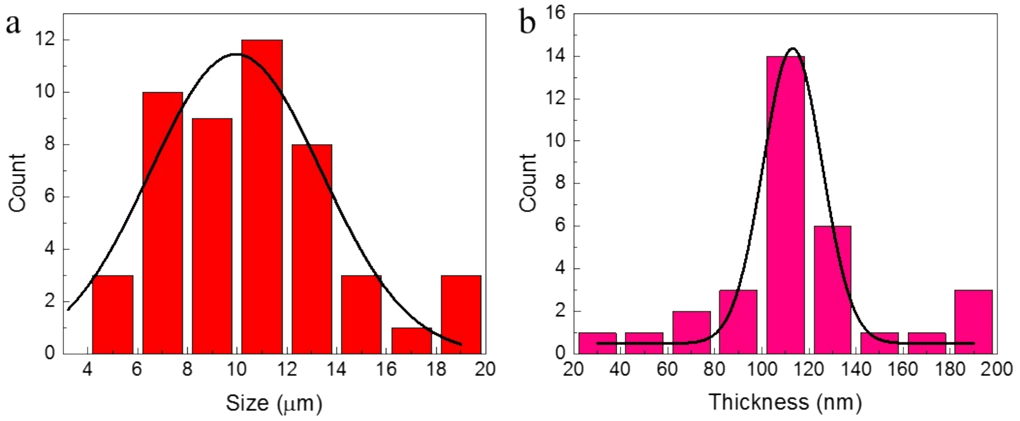

Fig. S1 The statistical data of the sizes (a) and thicknesses (b) of 2D perovskite microplates.

The average size and thickness of the microplates are 11.4 μm and 113 nm, respectively. Indeed, the size distribution of the microplates is relatively broad, but mostly in the

range of 7-15 μm. As contrast, their thicknesses are quite uniform.

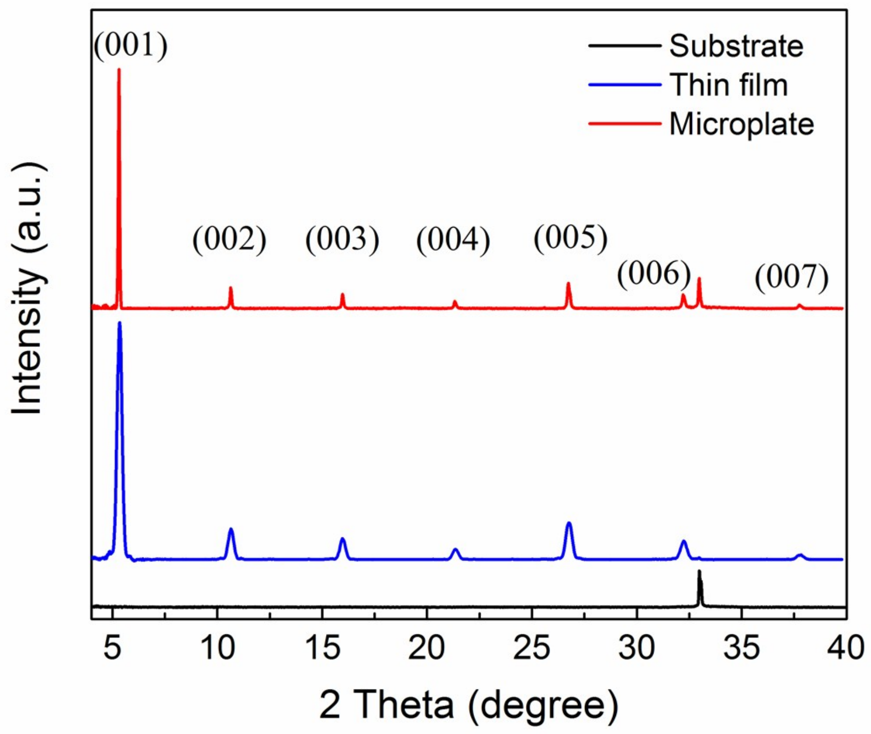

Fig. S2 XRD patterns of the samples before (blue line) and after (red line) acetone vapor treatment. The substrate data (black line) are presented for comparison.

Diffraction peaks at 5.33 °, 10.65 °, 15.97 °, 21.35 ° and 26.76 ° are assigned to the (001), (002), (003), (004) and (005) lattice planes of the layered $(PEA)_2PbBr_4$ structure, respectively.[1] The linewidth of the (001) peak for the samples after acetone vapor treatment is 0.0669 °, while that for the film samples is 0.2676 °. The peak at 32.98 ° originates from the Si/SiO_2 substrate.

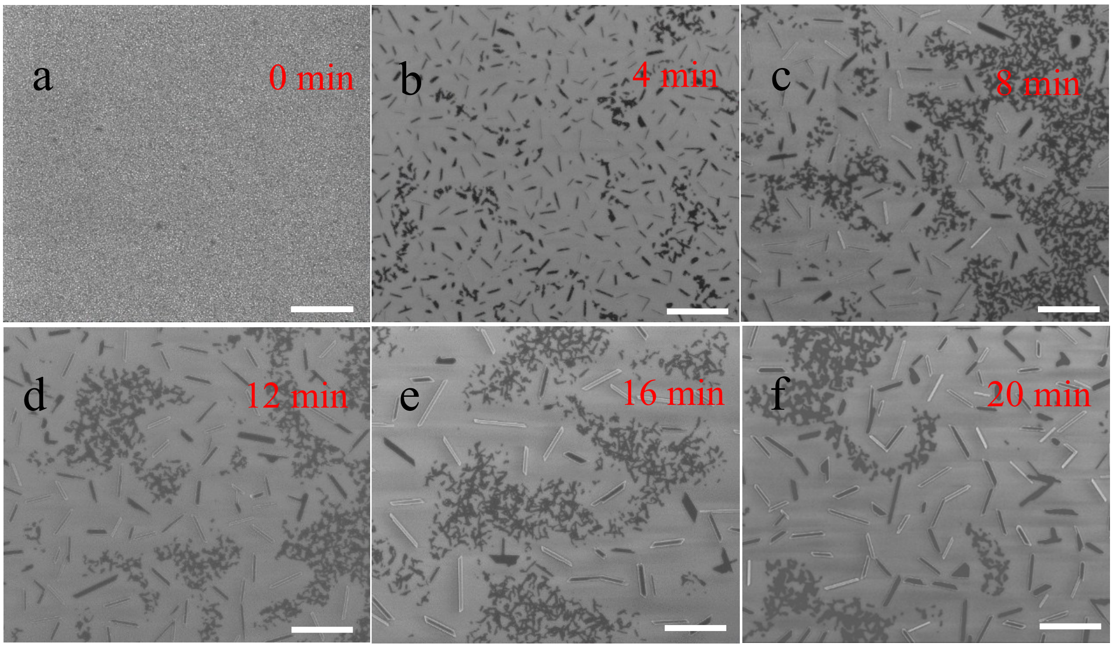

Fig. S3 SEM images of the growth process of $(PEA)_2PbBr_4$ microcrystals. All scale bars are 5 μm. (a) $(PEA)_2PbBr_4$ thin film without acetone vapor treatment. (b-f) $(PEA)_2PbBr_4$ microcrystals grown with acetone vapor treatment at room temperature for different time, corresponding to 4 min, 8 min, 12 min, 16 min and 20 min, respectively.

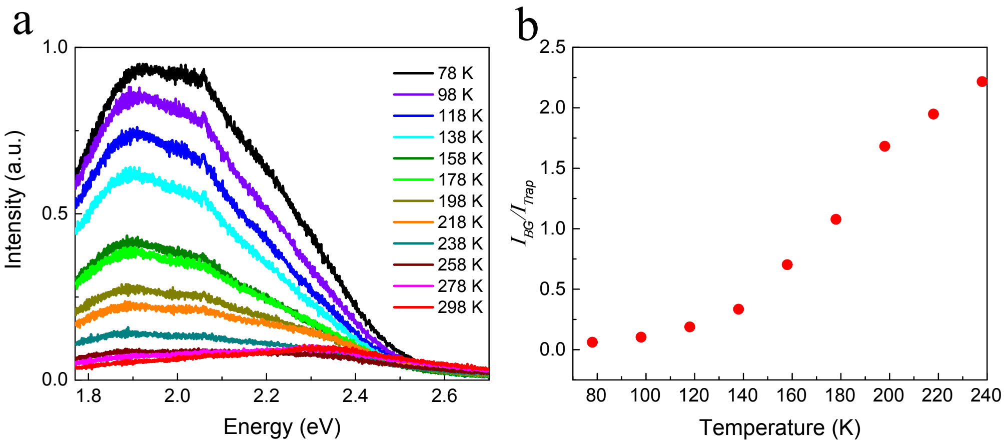

Fig. S4 (a) A magnification of temperature dependent PL spectroscopy ranging from 1.77 eV to 2.7 eV. (b) The ratio I_{BG}/I_{Trap} from 78 K to 238 K.

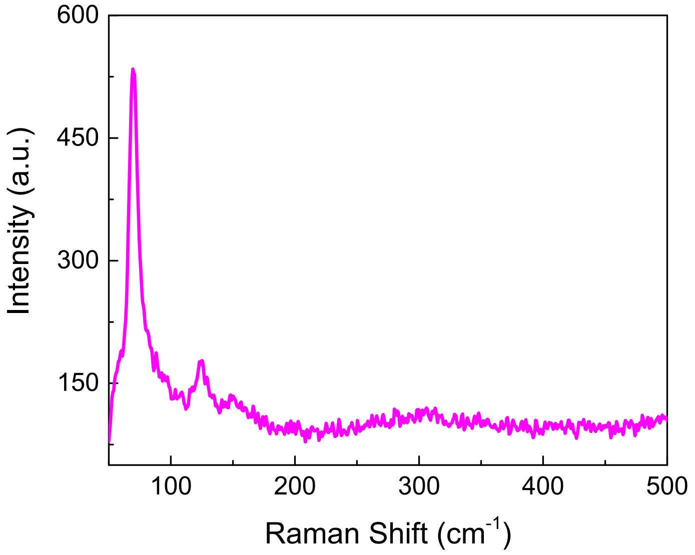

Fig. S5 Raman spectrum of the $(PEA)_2PbBr_4$ microplates.

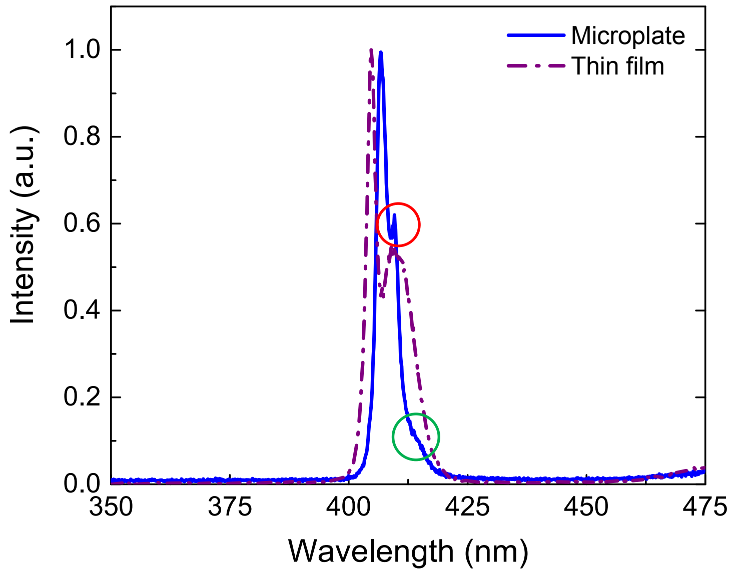

Fig. S6 PL spectra of the $(PEA)_2PbBr_4$ microplates (blue) and thin film (purple) at 78 K. More concentrated solution is used to grow the thin film to get enough signals for the low temperature measurement.

Table R1 Summary of the fitting results with the plot of linewidth

Parameters	Value	Standard error
Γ_0 (meV)	26	1
Γ_{op} (meV)	276	36
$\hbar\omega_{op}$ (meV)	50	3

References

1 D. Ma, Y. Fu, L. Dang, J. Zhai, I. A. Guzei and S. Jin, *Nano Res.*, 2017, **10**, 2117-2129.
